# A Versatile Gene Delivery System for Efficient and Tumor Specific Gene Manipulation in vivo

**DOI:** 10.15190/d.2016.5

**Published:** 2016-06-22

**Authors:** Wei Wang, Bingning Dong, Michael M. Ittmann, Feng Yang

**Affiliations:** Department of Molecular and Cellular Biology, Baylor College of Medicine, Houston, TX, 77030, USA; Department of Pathology and Immunology, Baylor College of Medicine, Houston, TX, 77030, USA

**Keywords:** RCAS, RCI, RCI-PyMT, RCI-Oncogene, TVA, Cre, knockout

## Abstract

The Replication-Competent Avian Sarcoma-leukosis virus long-terminal repeat with splice acceptor (RCAS)-Tumor Virus A (TVA) gene delivery system has been created based on the fact that avian sarcoma leukosis virus subgroup A only infects cells expressing its receptor, TVA. This system has been successfully applied to create various mouse models for human cancers. Here we briefly discuss the advantages and the potential caveats of using this RCAS-TVA gene delivery system in cancer research. We also introduce and discuss how our newly designed RCAS-based gene delivery system (RCI-Oncogene, for RCAS-Cre-IRES-Oncogene) allows concise and efficient manipulation of gene expression in tumors in vivo, and how this system can be used to rapidly study the biological function of gene(s) and/or the collaborative actions of multiple genes in regulating tumor initiation, progression and/or metastasis.

## 1. The RCAS-TVA gene delivery system

Avian sarcoma leukosis virus subgroup A (ASLV-A) only infects cells expressing its receptor, tumor virus A (TVA). Mammalian cells do not express TVA protein, therefore, are resistant to ASLV-A virus infection. Based on this, the replication-competent ASLV long-terminal repeat (LTR) with splice acceptor (RCAS)-TVA gene delivery system was developed. For this purpose, several genetically modified mouse lines expressing TVA in various specific cell types or tissues have been generated, including those targeting TVA expression in all tissues (β-actin promoter)^1^, epithelium (Keratin 19 promoter)^2^, Hepatocytes (Albumin promoter)^3^, mammary gland epithelium (MMTV promoter)^4^, endothelial cells (TIE2 promoter)^5^, and skeletal and cardiac smooth muscle cells (alpha-sk-actin promoter)^6^,* etc*. In addition, efforts have also been made to target TVA expression in cells with activation of a specific pathway, such as the TOP-*tva* transgenic model expressing TVA driven by the Wnt-responsive TOP promoter^7^. These TVA-expressing mouse lines are susceptible to infection by RCAS viruses for virus-mediated gene expression. This RCAS-TVA gene delivery system has, in theory, created endless opportunities in rapidly examining the biological function of gene(s) *in vivo*, as well as dissecting the functional roles of gene(s) in mouse models for human diseases, including cancers. Here we will briefly discuss the advantages and the potential caveats when using this RCAS-TVA system to model human cancers. We will also introduce our newly designed gene delivery system that allows concisely and efficiently studying the biological function of gene(s) in tumors *in vivo*.

## 2. The Advantages of RCAS-TVA system

The RCAS-TVA gene delivery system offers several advantages. An obvious immediate advantage is that this system provides a simple and much more cost-effective method to simultaneously study the biological function of multiple genes using a single TVA mouse strain. This is in comparison to the conventional transgenic / knockout / knockin methods that rely on creating individual genetically modified mouse lines and potentially multiple rounds of crossing. It may be especially important since recent years have witnessed the never-seen large set of data on genetic alterations in human cancer genomes. Frequently, it is extremely difficult to identify mutations/deletions/amplifications of driver and passenger gene in human cancers. In addition, it is labor-intensive and time-consuming to test the potential cooperative actions of multiple genes in tumorigenesis, tumor progression, and/or metastasis using the conventional transgenic / knockout / knockin approach. This RCAS-TVA gene delivery system may offer a unique opportunity to effectively address these questions, especially when using our newly designed delivery system as described below. Another potential usage of this system is to package cDNA or shRNA library into RCAS virus for systematical unbiased screening *in vivo*, although there is no published study on this yet. Furthermore, by tagging the specific types of cells, tissues and/or organs with expression of TVA and carefully selecting route of RCAS virus delivery, this gene delivery system allows concise targeting of specific cell types in a specific organ or tissue. RCAS virus will only effectively infect mouse lines expressing TVA, a great feature for the safety of lab animals and experimenters.

This RCAS-TVA model also provides a method for spatial and temporal gene expression *in vivo*, which is frequently very important for modeling human cancers using mouse models. In humans, cancers almost always arise from a population of single cells that acquired multiple genetic hits. These cell clones are physically separated from each other and are surrounded by normal cells. Therefore, it is very important to model this situation to understand how the cancer cells communicate with their surrounding normal cells and microenvironment, and how some cancer cell clones may jeopardize its surrounding normal cells and/or microenvironment for their own growth advantage. Furthermore, other than pediatric cancers, most human cancers are aging diseases. Therefore, it is crucial to model cancer in adult mice, and ideally in a controlled manner. This is especially important for studying human cancers known being affected by life events, such as the effects of pregnancy on human breast cancer^8^. Series of studies focusing on mammary tumors have provided an excellent example for utilizing the spatial and temporal gene expression offered by the RCAS-TVA system^4,7,9-12^. To specifically target mammary gland, MMTV-*tva* transgenic mouse line has been created**with**a “patchy” expression pattern of TVA in the mammary luminal epithelium^4^. Mammary tumors can be induced in the MMTV-*tva* mice by injection of the RCAS viruses expressing the polyomavirus middle T antigen (PyMT) into mammary gland via the nipple ducts. These mice develop mammary tumors that more closely recapitulate many features of human breast cancer than those from the MMTV-*PyMT* model. However, these mice developed lung metastasis at a much lower ratio compared with the MMTV-*PyMT *mice, making it not suitable for metastasis study^4,13^. Mammary tumor models can also be created using RCAS viruses expressing other oncogenes, such as a constitutively activated version of ErbB2/HER2/Neu (caErbB2). By intraductal injection of the RCAS-caErbB2 viruses into MMTV-tva mice 4-7 days before mating, this RCAS-TVA system was used to study the mechanisms underlying tumorigenesis of the pregnancy-associated breast cancers (PABCs), which are diagnosed during pregnancy, or within 5 years after parturition, in human^10^.

## 3. The potential caveats

Although there are clear advantages in using the RCAS-TVA gene delivery system in modeling human cancers in mouse, there are also potential caveats. One great limitation of the RCAS-TVA system comes from the limited capacity of the RCAS viruses. The size of the insert is in general limited to less than 3 kb, after which the viruses may lose its insert, produce truncations, and/or produce low-titer. The other limitation comes from the fact that RCAS viruses require cell division to incorporate viral cDNA into host genome. This may greatly limit the number of cells that can be infected in adult tissue and may create artificial effects by selectively infecting these dividing cells, which may not fully represent properties of the other quiescent cells.

## 4. Our novel gene delivery system for concise gene modification in tumors *in vivo*

The fibroblast growth factor receptor 1 (FGFR1) is frequently overexpressed in various types of human cancers. To define its functional contributions in prostate carcinoma, we have performed prostate-specific knockout of *fgfr1* in a well established spontaneous and metastatic prostate tumor model^14^. Three mouse strains including *fgfr1^loxP/loxP^* mice^15-17^, ARR_2_PBi-*Cre* mice^18^, and TRAMP mice^19^ were used to produce the target ARR_2_PBi-*Cre*/TRAMP/*fgfr1^loxP/loxP^* mice. This allows prostate-specific Cre expression (driven by ARR_2_PBi, an enhanced probasin promoter) for conditional knockout of *fgfr1* in TRAMP prostate tumors (induced by SV40 T/tag oncogenes driven by the probasin promoter). We demonstrated that ablation of *fgfr1* greatly inhibits primary tumor progression. Most importantly, the tumor cells that escaped *fgfr1* knockout gave rise to the poorly differentiated primary tumors and the metastatic tumors in the ARR_2_PBi-*Cre*/TRAMP/*fgfr1^loxP/loxP^* mice. This *fgfr1^KO^* escape phenotype indicates an essential role of FGFR1 in prostate tumor metastasis. Furthermore, this reveals that even the expression of Cre DNA recombinase (ARR_2_PBi-*Cre*) and T/tag oncogenes (TRAMP) were driven by essentially the same probasin promoter, they are not expressed in the identical cell populations. Some of the TRAMP prostate tumor cells (even a very small population) were never exposed to sufficient expression of Cre; due to an essential role of FGFR1 in driving prostate tumor progression and metastasis, these FGFR1 intact tumor cells expanded rapidly to give rise to the poorly differentiated primary tumors and provided seeds for metastasis. Finally, this *fgfr1^KO^* escape phenotype has greatly limited our ability to investigate the detailed molecular mechanisms underlying FGFR1 driving prostate tumor metastasis in this model.

**Figure 1:**
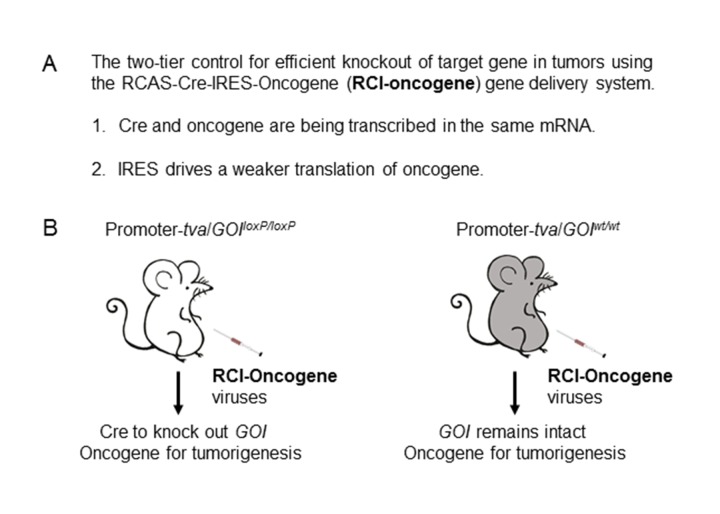
A. The two-tier control for efficient gene manipulation in our RCI-Oncogene/TVA gene delivery system B. Illustration of how RCI-Oncogene /TVA gene delivery system works

To both address the above issues and take advantage of the RCAS-TVA model, we have designed a new type of RCAS viral vector, the RCAS-Cre-IRES-Oncogene vector (named as RCI-Oncogene, IRES for internal ribosome entry site). In this case, Cre and oncogene are transcribed as one single mRNA, thus providing the first tier of control for concise expression of both genes in the same population of cells. This is extremely important for tumor modeling as discussed above. Furthermore, Cre protein expression will be driven by the regular 5' cap translation while oncogene protein expression will be driven by IRES-mediated translation (a weaker translation). This will provide the second tier of control for efficient Cre expression in oncogene-induced tumor cells. When RCI-Oncogene viruses are injected into the experimental TVA-mice carrying loxP flanked Gene of Interest (*GOI^loxP/loxP^*) and the control TVA-mice carrying *GOI^wt/wt^*, this allows high Cre expression to efficiently knock out *GOI *in oncogene-induced tumor cells (unpublished data;**Figure 1**).

We have tested and validated this model by generating the RCI-PyMT viruses and injecting them via nipple duct into the mammary glands of the control K19-*tva* mice expressing TVA in epithelium and the experimental K19-*tva/fgfr1^loxP/loxP^* mice. As expected, *fgfr1* remains intact in the control PyMT-induced mammary tumors, while *fgfr1* is ablated in the experimental tumors. Using this approach, we demonstrated that FGFR1 function in this RCI-PyMT induced mammary tumor model has an almost identical phenotype as our prostate cancer model^14^ (unpublished results).

Our new RCI-Oncogene model has brought several exciting opportunities. For example, by simply switching oncogenes in the RCI-Oncogene viruses (minimal work involves molecular cloning and packaging viruses), we can now easily assess how different oncogenes differentially cooperate with Cre-mediated loss of GOI (or overexpression of GOI as described below) to regulate tumor initiation, progression, and/or metastasis. This is in sharp contrast to the otherwise extremely time-consuming, costly, and labor-intensive works when conventional transgenic / knockout / knockin approaches are used. Furthermore, our RCI-Oncogene model will provide concise and efficient knockout or overexpression of target gene(s) in tumor cells, which may not be possible in most of the studies using the conventional transgenic / knockout / knockin approaches. In addition, our modified system may alter tumor progression and metastasis. For example, our RCI-PyMT mammary tumor model exhibits delayed tumor progression, which allows greatly increased lung metastasis (unpublished results). This is in contrast to the original RCAS-PyMT model that exhibits little lung metastasis^4^. Therefore, our RCI-PyMT mammary tumor model opened a new window for the study of genes that may be involved in tumor metastasis, which is extremely difficult to study when using the original RCAS-PyMT model.

Our model can also be easily adapted into different configurations. For example, we can use RCI-Oncogene model to specifically turn on the expression of GOI in mice carrying a transgene as "promoter-loxP-stop-LoxP-GOI"; in this case, Cre will specifically remove the loxP flanked transcription "stop" signal and efficiently turn on the expression of the GOI in the tumor cells. We can also study the function of a GOI in tumors using RCAS viruses with configuration of RCAS-GOI-IRES-Oncogene. Again, our designed two-tier expression control system will allow efficient expression of GOI in oncogene-induced tumors. Finally, we may use RCAS-GOI-IRES-Cre virus to knock out tumor suppressor gene such as *pten* and *p53*, and/or turn on oncogene carried by the host mice, such as K-Ras(G12D) in the *Kras^LSL-G12D^*mouse strain^20^. This will allow studying how GOI affects tumors induced by loss of tumor suppressor(s) or activation of oncogene(s). Again, our designed two-tier expression control system will allow efficient expression of GOI in tumors induced by loss of tumor suppressor(s) or overexpression of oncogene(s).

As previously discussed, one limitation of the RCAS-TVA gene delivery system is the limited capacity, which is typically less than 3 kb. Given the fact that Cre DNA recombinase cDNA is about 1.0 kb and IRES fragment is about 0.6 kb, this leaves about 1.4 kb as the maximum length of oncogene cDNA. This allows the incorporation of several well-documented and commonly used oncogenes or oncogenic proteins, including mutated Ras proteins (K-Ras, N-Ras, and H-Ras), C-Myc, N-Myc, and PyMT, *etc*. In addition, if necessary, DNA encoding for the 2A self-cleaving peptide sequence such as the thosea asigna virus 2A (T2A), foot-and-mouth disease virus 2A (F2A), equine rhinitis A virus 2A (E2A), and porcine teschovirus-1 2A (P2A)^21,22^ can be introduced to replace the IRES fragment, which will further increase the capacity of this vector. Lentivirus pseudotyped with the ALSV envelope protein has been used to infect none-dividing TVA expressing cells *in vivo *for tumor study^23^. These viruses can also handle larger insert; however the virus titers are usually low. Finally, the lentivirus has also been directly used to deliver oncogene expression into mice for tumor modeling and studies, such as the recent RapidCaP prostate tumor model^24-26^. However, without tagging any specific cell types, tissues, or organs, it remains debatable on how broadly this approach can be used for tumor modeling and studies without introducing significant artifacts.

## KEY POINTS

◊ **The RCAS-TVA gene delivery system provides a simple and rapid approach to study gene function in mouse models for human cancers.**

◊ **Our newly designed RCI-Oncogene (for RCAS-Cre-IRES-Oncogene) virus provides a versatile gene delivery system with precise two-tier control for accurate and efficient gene manipulation in tumors in vivo (unpublished results).**

◊ **It remains an open question whether this system can be successfully used for library-based in vivo screening.**

## References

[1] Federspiel M J, Swing D A, Eagleson B, Reid S W, Hughes S H (1996). Expression of transduced genes in mice generated by infecting blastocysts with avian leukosis virus-based retroviral vectors.. Proceedings of the National Academy of Sciences of the United States of America.

[2] Morton Jennifer P, Mongeau Michelle E, Klimstra David S, Morris John P, Lee Yie Chia, Kawaguchi Yoshiya, Wright Christopher V E, Hebrok Matthias, Lewis Brian C (2007). Sonic hedgehog acts at multiple stages during pancreatic tumorigenesis.. Proceedings of the National Academy of Sciences of the United States of America.

[3] Lewis Brian C, Klimstra David S, Socci Nicholas D, Xu Su, Koutcher Jason A, Varmus Harold E (2005). The absence of p53 promotes metastasis in a novel somatic mouse model for hepatocellular carcinoma.. Molecular and cellular biology.

[4] Du Zhijun, Podsypanina Katrina, Huang Shixia, McGrath Amanda, Toneff Michael J, Bogoslovskaia Ekaterina, Zhang Xiaomei, Moraes Ricardo C, Fluck Michele, Allred D Craig, Lewis Michael T, Varmus Harold E, Li Yi (2006). Introduction of oncogenes into mammary glands in vivo with an avian retroviral vector initiates and promotes carcinogenesis in mouse models.. Proceedings of the National Academy of Sciences of the United States of America.

[5] Montaner Silvia, Sodhi Akrit, Molinolo Alfredo, Bugge Thomas H, Sawai Earl T, He Yunsheng, Li Yi, Ray Patricio E, Gutkind J Silvio (2003). Endothelial infection with KSHV genes in vivo reveals that vGPCR initiates Kaposi's sarcomagenesis and can promote the tumorigenic potential of viral latent genes.. Cancer cell.

[6] Federspiel M J, Bates P, Young J A, Varmus H E, Hughes S H (1994). A system for tissue-specific gene targeting: transgenic mice susceptible to subgroup A avian leukosis virus-based retroviral vectors.. Proceedings of the National Academy of Sciences of the United States of America.

[7] Bu Wen, Zhang Xiang, Dai Hua, Huang Shixia, Li Yi (2013). Mammary cells with active Wnt signaling resist ErbB2-induced tumorigenesis.. PloS one.

[8] Schedin Pepper (2006). Pregnancy-associated breast cancer and metastasis.. Nature reviews. Cancer.

[9] Bu W, Chen J, Morrison G D, Huang S, Creighton C J, Huang J, Chamness G C, Hilsenbeck S G, Roop D R, Leavitt A D, Li Y (2011). Keratin 6a marks mammary bipotential progenitor cells that can give rise to a unique tumor model resembling human normal-like breast cancer.. Oncogene.

[10] Haricharan S, Hein S M, Dong J, Toneff M J, Aina O H, Rao P H, Cardiff R D, Li Y (2014). Contribution of an alveolar cell of origin to the high-grade malignant phenotype of pregnancy-associated breast cancer.. Oncogene.

[11] Holloway Kimberly R, Sinha Vidya C, Bu Wen, Toneff Michael, Dong Jie, Peng Yi, Li Yi (2016). Targeting Oncogenes into a Defined Subset of Mammary Cells Demonstrates That the Initiating Oncogenic Mutation Defines the Resulting Tumor Phenotype.. International journal of biological sciences.

[12] Haricharan Svasti, Dong Jie, Hein Sarah, Reddy Jay P, Du Zhijun, Toneff Michael, Holloway Kimberly, Hilsenbeck Susan G, Huang Shixia, Atkinson Rachel, Woodward Wendy, Jindal Sonali, Borges Virginia F, Gutierrez Carolina, Zhang Hong, Schedin Pepper J, Osborne C Kent, Tweardy David J, Li Yi (2013). Mechanism and preclinical prevention of increased breast cancer risk caused by pregnancy.. eLife.

[13] Du Zhijun, Li Yi (2007). RCAS-TVA in the mammary gland: an in vivo oncogene screen and a high fidelity model for breast transformation?. Cell cycle (Georgetown, Tex.).

[14] Yang Feng, Zhang Yongyou, Ressler Steven J, Ittmann Michael M, Ayala Gustavo E, Dang Truong D, Wang Fen, Rowley David R (2013). FGFR1 is essential for prostate cancer progression and metastasis.. Cancer research.

[15] Pirvola Ulla, Ylikoski Jukka, Trokovic Ras, Hébert Jean M, McConnell Susan K, Partanen Juha (2002). FGFR1 is required for the development of the auditory sensory epithelium.. Neuron.

[16] Trokovic Nina, Trokovic Ras, Mai Petra, Partanen Juha (2003). Fgfr1 regulates patterning of the pharyngeal region.. Genes & development.

[17] Trokovic Ras, Trokovic Nina, Hernesniemi Sanna, Pirvola Ulla, Vogt Weisenhorn Daniela M, Rossant Janet, McMahon Andrew P, Wurst Wolfgang, Partanen Juha (2003). FGFR1 is independently required in both developing mid- and hindbrain for sustained response to isthmic signals.. The EMBO journal.

[18] Jin Chengliu, McKeehan Kerstin, Wang Fen (2003). Transgenic mouse with high Cre recombinase activity in all prostate lobes, seminal vesicle, and ductus deferens.. The Prostate.

[19] Gingrich J R, Barrios R J, Morton R A, Boyce B F, DeMayo F J, Finegold M J, Angelopoulou R, Rosen J M, Greenberg N M (1996). Metastatic prostate cancer in a transgenic mouse.. Cancer research.

[20] Jackson E L, Willis N, Mercer K, Bronson R T, Crowley D, Montoya R, Jacks T, Tuveson D A (2001). Analysis of lung tumor initiation and progression using conditional expression of oncogenic K-ras.. Genes & development.

[21] Ryan M D, Drew J (1994). Foot-and-mouth disease virus 2A oligopeptide mediated cleavage of an artificial polyprotein.. The EMBO journal.

[22] Kim Jin Hee, Lee Sang-Rok, Li Li-Hua, Park Hye-Jeong, Park Jeong-Hoh, Lee Kwang Youl, Kim Myeong-Kyu, Shin Boo Ahn, Choi Seok-Yong (2011). High cleavage efficiency of a 2A peptide derived from porcine teschovirus-1 in human cell lines, zebrafish and mice.. PloS one.

[23] Siwko Stefan K, Bu Wen, Gutierrez Carolina, Lewis Brian, Jechlinger Martin, Schaffhausen Brian, Li Yi (2008). Lentivirus-mediated oncogene introduction into mammary cells in vivo induces tumors.. Neoplasia (New York, N.Y.).

[24] Cho Hyejin, Herzka Tali, Zheng Wu, Qi Jun, Wilkinson John E, Bradner James E, Robinson Brian D, Castillo-Martin Mireia, Cordon-Cardo Carlos, Trotman Lloyd C (2014). RapidCaP, a novel GEM model for metastatic prostate cancer analysis and therapy, reveals myc as a driver of Pten-mutant metastasis.. Cancer discovery.

[25] Cho Hyejin, Herzka Tali, Stahlhut Carlos, Watrud Kaitlin, Robinson Brian D, Trotman Lloyd C (2015). Rapid in vivo validation of candidate drivers derived from the PTEN-mutant prostate metastasis genome.. Methods (San Diego, Calif.).

[26] Nowak Dawid G, Cho Hyejin, Herzka Tali, Watrud Kaitlin, DeMarco Daniel V, Wang Victoria M Y, Senturk Serif, Fellmann Christof, Ding David, Beinortas Tumas, Kleinman David, Chen Muhan, Sordella Raffaella, Wilkinson John E, Castillo-Martin Mireia, Cordon-Cardo Carlos, Robinson Brian D, Trotman Lloyd C (2015). MYC Drives Pten/Trp53-Deficient Proliferation and Metastasis due to IL6 Secretion and AKT Suppression via PHLPP2.. Cancer discovery.

